# Semiautomatic robust regression clustering of international trade data

**DOI:** 10.1007/s10260-021-00569-3

**Published:** 2021-06-11

**Authors:** Francesca Torti, Marco Riani, Gianluca Morelli

**Affiliations:** 1 European Commission, Joint Research Centre (JRC), Via Enrico Fermi 2749, 21027 Ispra, VA Italy; 2 Dipartimento di Scienze Economiche e Aziendali and Interdepartmental Centre for Robust Statistics, University of Parma, Via Kennedy 6, 43125 Parma, Italy

**Keywords:** TCLUST, Forward search, Regression, Clustering, Trimming, Outliers, Multiple start, Monitoring, International trade

## Abstract

The purpose of this paper is to show in regression clustering how to choose the most relevant solutions, analyze their stability, and provide information about best combinations of optimal number of groups, restriction factor among the error variance across groups and level of trimming. The procedure is based on two steps. First we generalize the information criteria of constrained robust multivariate clustering to the case of clustering weighted models. Differently from the traditional approaches which are based on the choice of the best solution found minimizing an information criterion (i.e. BIC), we concentrate our attention on the so called optimal stable solutions. In the second step, using the monitoring approach, we select the best value of the trimming factor. Finally, we validate the solution using a confirmatory forward search approach. A motivating example based on a novel dataset concerning the European Union trade of face masks shows the limitations of the current existing procedures. The suggested approach is initially applied to a set of well known datasets in the literature of robust regression clustering. Then, we focus our attention on a set of international trade datasets and we provide a novel informative way of updating the subset in the random start approach. The Supplementary material, in the spirit of the Special Issue, deepens the analysis of trade data and compares the suggested approach with the existing ones available in the literature.

## Motivations and contributions

The purpose of this paper is to provide the user with a set of semiautomatic tools in the context of regression clustering which can help to select the optimal number of groups (or more generally to find a set of relevant solutions), give insights about the optimal restriction factors among the variances of the estimated residual variances and finally enable to estimate the optimal trimming level keeping into account that it can depend on the chosen solution.

The above issues are of paramount importance in the context of international trade data because in this case particular groups can be associated with potential illegal behaviour. The detection of fraudulent behaviour in economic activities is more and more central in the political priorities of governments. In the EU, the European Commission (EC) has taken strong commitments in this regard (von der Leyen 2019), stressing the importance of the fight against tax fraud, money laundering and customs fraud. In relation to the last priority, the EC intends to “reinforce customs risk management and support effective controls by the Member States”. This requires a harmonized (if not unified) approach to the problem but, before anything else, it is necessary to dispose of analytic instruments appropriate to the variety and amount of data collected by the administrations. One of these data sources are the declarations made at the Customs by the importers of goods in the EU, which are transferred to the EC every day. We have a long tradition in analyzing these and derived data with instruments developed in support to the work of Customs and EC law enforcement services. For example, in Perrotta and Torti (2010) we approached the problem of detecting price outliers in regression on monthly aggregates of traded values and quantities, in Riani et al. (2018) we addressed a related price estimation problem complicated by potential small sample size issues, while in Rousseeuw et al. (2019) we also considered the detection of anomalies in time series of such trade flows. Perhaps the most important limitation of these and other works is that they assume rather homogeneous trade flows, with a dominant population possibly affected by a certain amount of contamination, while for many commodities the data show a variety of possible sub-populations characterized by different prices or weights per unit. For example, the three types of filtering facepiece (FFP) mask for respiratory protection and other classical surgical masks that have become famous during the COVID-19 crisis, currently fall in the Combined Nomenclature under code 6307.90.98 together with other products, but these products clearly differ for both price and specific weight (the grammage for the FFP masks is at least $200\;{\mathrm{g/m}}^2$ while the surgical masks have lower weight). Currently the Customs are supposed to check their import/export carefully and a data-driven instrument to promptly distinguish their declarations would help them a lot.

We naturally address these heterogeneity problems from a *clusterwise linear regression* perspective, in a robust setting to account for the ubiquitous contamination problem. Differently from the traditional approaches in which the choice of the optimal number of groups is associated with the minimization of an information criterion based on a unconstrained likelihood (e.g. Fraley and Raftery 2002), in this paper we focus our attention on the so called optimal stable solutions, that is solutions which do not depend on a specific choice of a constraining parameter. In the context of international trade this is of paramount importance, because it is essential to show that a particular classification can still be found in presence of minor changes of the input parameters.

The structure of the paper is as follows. In Sect. 2 we introduce the notation and illustrate the methodological problems we are trying to address. In Sect. 3 we describe our procedure. The approach is *semi*-automatic because, although the best solutions are identified in an automatic way by comparing a set of different parameter combinations, a proper inferential test to assess the different solutions is not yet available.

In Sect. 4 we introduce a motivating example about the European Union trade of face masks which shows that the application of traditional approaches completely fails to extract the most relevant features.

In Sect. 5 we show the practical applications of the new tools for the choice of the level of trimming, restriction factor and number of groups. The suggested approach is initially applied to a set of well known real and contaminated datasets taken from the specialized literature on regression clustering and, in the Supplementary Material, to datasets that mimic the typical shape of international trade data, with different levels of overlap and amount of contamination. Section 6 concludes and provides food for thought for additional research. The paper contains an appendix where we prove a theorem about the optimal updating in the forward search applied to international trade data.

The results in this work can be easily replicated using functions and data that we have integrated in our Flexible Statistics for Data Analysis software package, the FSDA toolbox for MATLAB, which is available as “Add-On” inside MATLAB or in github (for interested contributors or experimenters: https://github.com/UniprJRC/FSDA). The extensive documentation, available at http://rosa.unipr.it/FSDA.html, simplifies the replication of the results in this paper and the extension to other datasets and problems.

## Methodology

In this section we describe the methodological problem we are trying to address, introduce the notation and the relevant references. Let the vector of covariates *X* (which might be univariate or multivariate) and the response variable *Y* be defined on $\varOmega$ with values in ${{{\mathcal {X}}}} \times {{{\mathcal {Y}}}}$ $\subseteq R^{p-1} \times R$. Then, $\{x_i, y_i\}$, $i=1, 2, \ldots , n$, represents a i.i.d. random sample of size *n* drawn from (*X*, *Y*). If we suppose that $\varOmega$ can be partitioned into *k* groups, say $\varOmega _1$, $\varOmega _2$, $\ldots$, $\varOmega _k$, the general formulation of the regression clustering mixture model has a density which can be written as

1 $$\begin{aligned} p(x,y, \theta ) = \sum _{g=1}^k p(y|x, \theta _{y,g}) p(x, \theta _{x,g}) \pi _g, \end{aligned}$$

where $p(y|x, \theta _{y,g})$ is the conditional density of *Y* given *x* in $\varOmega _g$ which depends on the vector of parameters $\theta _{y,g}$, $p(x, \theta _{x,g})$ is the marginal density of *X* in $\varOmega _g$ which depends on the vector of parameters $\theta _{x,g}$, and $\pi _g$ reflects the importance of $\varOmega _g$ in the mixture with the usual constraints $\pi _g>0$ and $\sum _{g=1}^k \pi _g=1$. Vector $\theta$ denotes the full set of parameters $\theta =(\theta _{y,g}^T \; \theta _{x,g}^T)^T$. It is customary to assume that in each group *g* the conditional relationship between *Y* and *x*, $p(y|x, \theta _{y,g})$, can be written as

2 $$\begin{aligned} Y= \beta _{0,g}+x^T \beta _g + \epsilon _g, \end{aligned}$$

where $\epsilon _g \sim N(0, \; \sigma ^2_g)$, and $\beta _g$ and $\sigma _g$ are respectively the $(p-1) \times 1$ vector of regression parameters ($\beta _{1,g}, \beta _{2,g}, \ldots , \beta _{p-1,g})^T$ and scale parameter referred to component *g*. This formulation was originally proposed by Gershenfeld (1997) and was developed in the context of media technology, in order to build a digital violin. With the linearity and normality assumption, the first two conditional moments of *Y* given *x* can be written as $E(Y|x, \beta _{0g}, \beta _g, \sigma _g)=\beta _{0,g}+x^T \beta _g$, $var(Y|x, \beta _{0,g}+\beta _g, \sigma _g)=\sigma _g^2$ . If, in addition, we also assume that the *X* distribution is multivariate normal, that is

3 $$\begin{aligned} p(x, \theta _{x,g})= \phi _{p-1}(x, \mu _g, \varSigma _g), \end{aligned}$$

where $\phi _{p-1}(x, \mu _g, \varSigma _g)$ denotes the density of a $p-1$-variate Gaussian distribution, with mean vector $\mu _g$ and covariance $\varSigma _g$, model (1) becomes the so called linear Gaussian Cluster Weighted Model (CWM) (Gershenfeld et al. 1999) and can be written as

4 $$\begin{aligned} p(x, y, \theta )= \sum _{g=1}^k \phi (y; \beta _{0,g}+ \beta _g^T x, \sigma _g^2) \phi _{p-1}(x, \mu _g, \varSigma _g) \pi _g. \end{aligned}$$

It is interesting to notice that clustering around regression (DeSarbo and Cron 1988) can be seen as a special case of equation (4) by setting $\phi _{p-1}(x, \mu _g, \varSigma _g)=1$, that is ignoring the distribution of *X*.

Equation (4) corresponds to a mixture of regressions with weights $\phi _{p-1}(x, \mu _g, \varSigma _g)$ depending not only on $\pi _g$, but also on the covariate distribution in each component *g*. This leads to define the following log-likelihood function to be maximized (mixture log-likelihood $L_{Mixt}(\theta )$)

5 $$\begin{aligned} L_{Mixt}(\theta )=\sum _{i=1}^n \log \left[ \sum _{g=1}^k \phi (y_i|b_{0,g}, x_i^T b_g , s_g^2) \phi _{p-1}(x_i, m_g, S_g) p_g \right] , \end{aligned}$$

where $\theta =(p_1,...,p_k, b_{0,1}, \ldots , b_{0,k}, b_1, \ldots , b_k, s_1^2, \ldots , s_k^2, m_1,...,m_k,S_1,..., S_k)$ is the set of parameters satisfying $p_g\ge 0$ and $\sum _{g=1}^k p_g=1$, $b_g \in {R}^{p-1}$, $b_{0,g} \in {R}^{1}$, $s_g^2 \in {R}^+$, $m_j\in {R}^{p-1}$ and $S_j$ a positive semi-definite symmetric $(p-1)\times (p-1)$ matrix. The optimal set of parameters based on this likelihood is

6 $$\begin{aligned} {\widehat{\theta }}_{\text {Mixt}}=\arg \max _{\theta }L_{\text {Mixt}}(\theta ). \end{aligned}$$

Once ${\widehat{\theta }}_{\text {Mixt}}=({\widehat{p}}_1,..., {\widehat{p}}_k, {{\widehat{b}}}_{0,1}, \ldots , {{\widehat{b}}}_{0,k}, {\widehat{b}}_1,..., {\widehat{b}}_k, {\widehat{s}}_1^2,..., {\widehat{s}}_k^2, {\widehat{m}}_1,..., {\widehat{m}}_k,{\widehat{S}}_1,..., {\widehat{S}}_k)$ is obtained, the observations in the sample are divided into *k* clusters by using posterior probabilities. That is, observation $(x_i \; y_i)$ is assigned to cluster *g*, if $g=\arg \max _{l} \phi (y_i| {{\widehat{b}}}_{0,l}, {{\widehat{b}}}_l^T x, {{\widehat{s}}}_l^2) \phi _{p-1}(x_i;{\widehat{m}}_l,{\widehat{S}}_l) {\widehat{p}}_l$.

In the so-called classification framework of model based clustering, the classification log-likelihood ($L_{\text {Cla}}(\theta )$) to be maximized is defined as

7 $$\begin{aligned} L_{\text {Cla}}(\theta )=\sum _{i=1}^n \sum _{g=1}^k z_{ig}(\theta ) \log \phi (y_i|b_{0g}, x_i^T b_g , s_g^2) \phi _{p-1}(x_i, m_g, S_g) p_g, \end{aligned}$$

where $$\theta =(p_1,...,p_k, b_{0,1}, \ldots , b_{0,k}, b_1, \ldots , b_k, s_1^2, \ldots , s_g^2, m_1,...,m_k,S_1,..., S_k)$$ and

$$\begin{aligned} z_{ig}(\theta )=\left\{ \begin{array}{l} 1\text { if } g=\arg \max _{l} \phi (y_i| {{\widehat{b}}}_{0,l}, {{\widehat{b}}}_l^T x, {{\widehat{s}}}_l^2) \phi _{p-1}(x_i;{\widehat{m}}_l,{\widehat{S}}_l) {\widehat{p}}_l, \qquad l=1, 2, \ldots , k, \\ 0\text { otherwise}. \end{array} \right. \end{aligned}$$

In this case, the optimal set of parameters is

8 $$\begin{aligned} {\widehat{\theta }}_{\text {cla}}=\arg \max _{\theta }L_{cla}(\theta ) \end{aligned}$$

and the observation $(x_i\; y_i)$ is now classified into cluster *g* if $z_{ig}({\widehat{\theta }}_{\text {Clas},g})=1$.

The target functions (5) and (7) are unbounded when no constraints are imposed on the scatter parameters. It is necessary therefore to impose constraints on the maximization on the set of eigenvalues $\{\lambda _r({\hat{S}}_g)\}$, $r=1, \ldots , (p-1)$, of the scatter matrices ${\hat{S}}_g$ by imposing

$$\begin{aligned} \lambda _{l_1} ({\hat{S}}_{g_1}) \le c_X \lambda _{l_2} ({\hat{S}}_{g_2}) \;\; \text{ for } \text{ every } \;\; 1 \le l_1 \ne l_2 \le p-1 \;\; \text{ and } \;\; 1 \le g_1 \ne g_2 \le k \end{aligned}$$

and to the variances ${\hat{s}}_g^2$ of the regression error terms, by requiring

$$\begin{aligned} {\hat{s}}_{g_1}^2 \le c_y {\hat{s}}_{g_2}^2 \qquad \text{ for } \text{ every } \qquad 1 \le g_1 \ne g_2 \le k . \end{aligned}$$

The constants $c_X \ge 1$ and $c_y \ge 1$ are real numbers (not necessarily equal) which guarantee that we are avoiding the cases $|{\hat{S}}_g| \rightarrow 0$ and $s^2_g \rightarrow 0$. Following (Cerioli et al. 2018), we consider the following values of the restriction parameters $2^0, 2^1, \ldots , 2^7$ because it enables us to consider a sharp grid of values close to 1.

As suggested by one of the referees, beyond the constraints on the scatter parameters, it is sometimes useful to impose on the vector of regression coefficients a homogeneous slope. In other words, instead of assuming that all regression parameters are class dependent, we can consider a mixture model whose components only differ in the intercept term i.e. $(\beta _{0,1}, \beta _{1,d}, \ldots , \beta _{p-1,d})^T$ $\ldots$ $(\beta _{0,k}, \beta _{1,d}, \ldots , \beta _{p-1,d})^T$. In Sect. 5.2 we test this option.

In the literature of robust regression it is widely known the effect of both vertical outliers in *Y* and outliers in *X*. Robustness can be achieved by discarding in each step of the maximization procedure a proportion of units equal to $\alpha$, associated with the smallest contributions to the target likelihood. More precisely, for example in the mixture modeling context, the Trimmed Cluster Weighted Model (TCWM) parameter estimates are based on the maximization of the following trimmed likelihood function $L_{\text {Mixt}}(\theta |\alpha , c_y, c_X)$ (García-Escudero et al. 2017)

9 $$\begin{aligned} L_{\text {Mixt}}(\theta |\alpha , c_y, c_X)=\sum _{i=1}^n z^*(x_i, y_i) \log \left[ \sum _{g=1}^k \phi (y_i| b_{0,g}, b_g^T x, s_g^2) \phi _{p-1}(x_i, m_g, S_g) p_g \right] , \end{aligned}$$

where $z^*(\cdot , \cdot )$ is a 0-1 trimming indicator function which tells us whether observation $(x_i \; y_i)$ is trimmed off ($z^*(x_i \; y_i)=0$) or not $(z^*(x_i \; y_i)=1$). A fixed fraction $\alpha$ of observations can be unassigned by setting $\sum _{i=1}^n z(x_i \; y_i)=[n(1-\alpha )]$. TCLUST-REG (García-Escudero et al. 2010b) can be considered as a particular case of TCWRM in which the contribution to the likelihood of $\phi _{p-1}(x_i, m_g, S_g)$ is set equal to 1.

However, if the component $\phi _{p-1}(x_i, m_g, S_g)$ is discarded, $\alpha$ just protects against vertical outliers in *Y*, since these data points have small $\phi (y_i| b_{0,g}, b_g^T x, s_g^2) p_g$ values, but it has no effect in diminishing the effect of outliers in the *X* space. Therefore, if we adopt a TCLUST-REG approach, it is necessary to consider (as done by García-Escudero et al. 2010b) a second trimming step, which discards a proportion $\alpha _X$ of the units, after taking into account their degree of remoteness in the X space, among the observations which have survived the first trimming operation. The original solution in TCLUST-REG was to fix $\alpha _X$ in advance, although there is no established indication of the link between this proportion and the breakdown properties of the methodology. Torti et al. (2018) have proposed to select $\alpha _X$ adaptively from the data using a multivariate outlier detection procedure in the space of the explanatory variables. The observations surviving to the two trimming steps are then used for updating the regression coefficients, weights and scatter matrices. This modification of the algorithm is usually referred in the literature as *adaptive TCLUST-REG*.

Torti et al. (2018) have also assessed how the performances of TCWM change in presence of possible misspecification of the distribution of the explanatory variables. Their experience is that the superior performance of TCWM can degenerate if the explanatory variable distribution is miss-specified and in this case the TCLUST-REG solution is preferable. On the other hand, Barabesi et al. (2016) have shown that the Tweedie distribution is a flexible model for describing the traded quantities in international trade data, but their work has also highlighted the complexity of developing efficient algorithms for estimating the Tweedie parameters with international trade data. In particular, the estimation method is not sufficiently flexible to address the thousands of different cases that the anti-fraud context described in this paper can potentially cover. For this reason, this work starts to describe the general context of TCWM as the appropriate framework of our anti-fraud context, while addressing the model choice issues in the simplified TCLUST-REG case.

An additional alternative approach to deal with contamination is the use of weighted likelihood estimation as done in Greco et al. (2020). In this approach it is necessary to select a smoothing parameter which controls the trade-off between efficiency and robustness. In this paper, for one of the datasets, we compare our results with those based on this approach.

## The suggested procedure

In this section we describe our two-step procedure for finding:

1. the optimal number of groups *k*;
2. the amount of first trimming level $\alpha$;
3. the optimal restriction factor $c_y$, among the variance of the error components or the scatter matrices of the covariates.

First we estimate one or more reasonable set of combinations of values of $c_y$ and *k*, given a large value of $c_X$ if TCWM is used (Sect. 3.1) and then we find the optimal trimming level (Sect. 3.2) through a monitoring approach (Riani et al. 2014; Cerioli et al. 2017 and more recently Torti et al. 2021). The choice of the best number of groups and/or the validation of a particular *k* can also be supported by a confirmatory forward search approach (Sect. 3.3).

### Preliminary estimate of restriction factor and number of groups

The choice of the optimal number of groups *k* can be done using an information criterion. Extending what has been done for multivariate analysis in the paper (Cerioli et al. 2018), we have 3 possibilities.

$$\begin{aligned} \text {MIX-MIX} : k_{\text {opt}}(c_y, c_X|\alpha )= & {} \arg \min _k \left\{ -2 L_{\text {Mixt,k}} ({\widehat{\theta }}_{\text {Mixt,k}}|\alpha , c_y, c_X)+v_k^{c_y, c_X} \right\} \\:= & {} \arg \min _k F_{MM}(k, c_y, c_X| \alpha ) \\ \text {MIX-CLA} : k_{\text {opt}}(c_y, c_X|\alpha )= & {} \arg \min _k \left\{ -2 L_{\text {Cla,k}} ({\widehat{\theta }}_{\text {Mixt,k}} |\alpha , c_y, c_X ) +v_k^{c_y, c_X} \right\} \\:= & {} \arg \min _k F_{MC}(k, c_y, c_X| \alpha ) \\ \text {CLA-CLA} : k_{\text {opt} }(c_y, c_X|\alpha )= & {} \arg \min _k \left\{ -2 L_{\text {Cla,k}} ({\widehat{\theta }}_{\text {Cla,k}} |\alpha , c_y, c_X ) +v_k^{c_y, c_X} \right\} \\:= & {} \arg \min _k F_{CC}(k, c_y, c_X| \alpha ) \end{aligned}$$

where $v_k^{c_y, c_X}$ is a penalty term defined as

$$\begin{aligned}&v_k^{c_y, c_X}= pk +(k-1)\\&\quad \qquad + (k-1) (1-1/c_y) +1 + 0.5p_1(p_1-1)k + (p_1 k-1) (1-1/c_X) +1. \end{aligned}$$

where $p_1 = p-1$. In our notation, “MIX-MIX” corresponds to the use of the Bayesian Information Criterion (BIC) (see, e.g., Fraley and Raftery 2002), while “MIX-CLA” corresponds to the use of the Integrated Complete Likelihood (ICL) method proposed by Biernacki et al. (2000). If $c_y \rightarrow \infty$ the ratio of the variances of the residuals becomes unconstrained. The same things happens to the the $\varSigma _X$ scatter matrices when $c_X \rightarrow \infty$.

#### Remark

With the constraint of common slopes it is necessary to replace the first term in the sum *pk* (number of regression parameters) of the different Information Criteria with $k+p_1$.

The plot which shows the values of the Information Criterion (IC) as function of *k*, that we call *elbow plot*, is generally used to find the appropriate number of groups. In most cases however this trajectory is a monotonic function of *k* (an example of this situation is shown in Sect. 4). In the context of constrained TCWM the situation is complicated by the fact that there are different trajectories, each associated with a combination of values of the restriction factors. In some simple cases all the trajectories follow the same pattern and therefore the best solution, independently from the restriction factor values, is easy to identify: this scenario is well represented in the case study of Sect. 5.1. In more complex cases, different trajectories may follow different paths, an example of this case is presented in Sect. 5.2. In addition, the elbow plot does not provide any information about the stability of the solutions as function of $c_y$ ($c_X$) or *k*.

It is important to remark that, differently from the traditional approach which is based on the minimization of a particular unconstrained information criterion, in this paper we focus our attention on the so called optimal stable solutions, that is the solutions which do not depend on a specific choice of a constraining parameter. In this paper we extend the graphical tool known as car-bike plot introduced by Cerioli et al. (2018) to the context of regression clustering and to the case of trimmed likelihood in order to select and visualize a ranked list of “optimal” choices for the pair $(k,c_y)$. The procedure first detects a list with *L* “plausible” partitions. Such “plausible” partitions may include some solutions that are essentially the same as others already detected, because spurious clusters made up with few almost collinear or very concentrated data points are found. In a second step, the partitions including repetitive solutions are discarded and we end up with a (typically very) reduced and ranked list with *T* “optimal” (non repetitive) partitions.

More formally, given a triple $(k,c_y,c_X)$, let ${\mathcal {P}}(k,c_y, c_X)$ denote the partition into *k* subsets which is obtained by solving the problem (5) or (7), with the given *k*, $c_X$ and $c_y$. Let us consider the sequence $k=1,...,K$, where *K* is the maximal number of clusters, and a sequence $c=c_1,...,c_C$ of *C* possible constraint values. For instance, the sequence of powers of 2, $c_1=2^0, c_2=2^1,..., c_C=2^{C-1}$ is recommended because it enables us to consider a sharp grid of values close to 1 (in this paper $C=8$).

In the context of TCWM in order to prevent the presence of spurious solutions in the *X* space we suggest to fix $c_X$ to the a finite large value (in this paper $\max (c_X)=128$). Once all the other parameters have been estimated it is possible to refine this value using the monitoring approach and the information criteria (MIX-MIX, MIX-CLA, CLA-CLA), given at the beginning of this section. If on the other hand, the data are highly non normal (as in the case of international trade data) we suggest to use TCLUST-REG with flexible second level of trimming as described in Torti et al. (2018). In what follows in order to avoid a cumbersome notation with symbol *c* we denote $c_y$ (with $c_X$ fixed at 128 in case of TCWM). Let $\text {ARI}({\mathcal {A}},{\mathcal {B}})$ denote the adjusted ARI index between partitions ${\mathcal {A}}$ and ${\mathcal {B}}$. We consider that two partitions ${\mathcal {A}}$ and ${\mathcal {B}}$ are “essentially the same” when $\text {ARI}({\mathcal {A}},{\mathcal {B}})\ge \varepsilon$, for a fixed threshold $\varepsilon$ (in this paper $\varepsilon =0.7$). Clearly, the higher the value of the threshold the greater is the number of tentative different solutions which are considered.

By using this notation, the proposed automated procedure may be described as follows:

1. *Obtain the list of “plausible” solutions*:
  1.1 *Initialize:* Start with $K\times C$ possible (*k*, *c*) pairs to be explored. Let ${\mathcal {E}}_0=\{(k,c):k=1,...,K\text { and }c=c_1,...,c_C\}$.
  1.2 *Iterate:* If ${\mathcal {E}}_{l-1}$ is the set of pairs (*k*, *c*) not already explored at stage $l-1$, then:
    1.2.1 Obtain $(k_{*}^l,c_{*}^l)=\arg \min _{(k,c)\in {\mathcal {E}}_{l-1}} F_m(k,c,c_X).$ where ($m=MM$, *MC* or *CC*.) For each “optimal” pair $(k_{\text {opt}}^t,c_{\text {opt}}^t)$, we analyze the so-called “best interval” ${\mathcal {B}}_t$ that is the set of consecutive values of *c* adjacent to $c_{\text {opt}}^t$ (say $c^*$) for which the solution remains optimal. That is:
      10 $$\begin{aligned} {\mathcal {B}}_t= & {} \{ c^*: F_m( k_{\text {opt}}^t,c^*) \le F_m(k_1,c_1)\} \nonumber \\ \text {where}= & {} (k_1,c_1) \in ( {\mathcal {E}}_{l-1} \cap k_1 \ne {k_\text {opt}}). \end{aligned}$$
      and the so-called “stable interval” defined as
      11 $$\begin{aligned} {\mathcal {S}}_t=\{ c: \text {ARI}( {\mathcal {P}}(k_{\text {opt}}^t,c) , {\mathcal {P}}(k_{\text {opt}}^t,c_{\text {opt}}^t) ) \ge \varepsilon \}. \end{aligned}$$
      A large interval ${\mathcal {B}}_t$ means that the number of clusters $k_{\text {opt}}^t$ is “optimal” in the sense of (10) for a wide range of *c* values. A large interval ${\mathcal {S}}_t$ means that the solution is “stable” in the sense of (11) because the change when moving *c* in that interval is irrelevant.
    1.2.2 Remove all cluster partitions $(k,c)\in {\mathcal {B}}_t \cup {\mathcal {S}}_t$ (set of similar partitions). Take ${\mathcal {E}}_{l}$ as the set ${\mathcal {E}}_{l-1}$ after removing the pairs yielding “similar” partitions found at step *l*.

1.3 *Finalize:* The iterative procedure ends when ${\mathcal {E}}_{L}=\emptyset$ (or when *L* is a positive prefixed integer number) and it returns $\{ (k_{*}^1,c_{*}^1) , (k_{*}^2, c_{*}^2), ..., (k_{*}^L,c_{*}^L) \}$ as a list with *L* “feasible” parameters combinations.

2. *Obtain the list of “optimal” (non repetitive) solutions:*

2.1 *Initialize:* Start from ${\mathcal {I}}_0=\{1,...,L\}$ and the $L\times L$ matrix $(d_{r,s})_{r,s=1,...,L}$, where
  $$\begin{aligned} d_{r,s}= \text {ARI}( {\mathcal {P}}(k_{*}^r,c_{*}^r) , {\mathcal {P}}(k_{*}^s,c_{*}^s) . ), \end{aligned}$$
2.2 *Iterate:* Given ${\mathcal {I}}_{t-1}$ the non discarded “plausible” solutions at stage $t-1$:
  2.2.1 Take $(k_{\text {opt}}^t,c_{\text {opt}}^t)=(k_{*}^{l_t},c_{*}^{l_t})$ where $l_t$ is the *t*-th element of ${\mathcal {I}}_{t-1}$ (where the indexes in ${\mathcal {I}}_{t-1}$ are sorted from lowest to highest).
  2.2.2 Discard “repetitive” solutions (i.e., those that are similar to the already detected “optimal” ones): ${\mathcal {I}}_t={\mathcal {I}}_{t-1}\setminus \{r: r\in {\mathcal {I}}_{t-1}, r> l_t \text { and } d_{r,l_t}\ge \varepsilon \}.$
2.3 *Finalize:* The iterative procedure ends when ${\mathcal {I}}_{T}=\emptyset$. It returns
  $$\begin{aligned} \{ (k_{\text {opt}}^1,c_{\text {opt}}^1) , (k_{\text {opt}}^2, c_{\text {opt}}^2), ..., (k_{\text {opt}}^T,c_{\text {opt}}^T) \} \end{aligned}$$
  as the “optimal” pairs.

The results of the procedure can be visualized in an informative plot known as *car-bike plot* (for an example see Fig. 14). In the car-bike plot the optimal pairs are shown with circles (“bikes”). In the circle we write two integers that rank the solution’s quality. More precisely, the first integer indicates the rank of the solution among the optimal non-repetitive ones, while the second integer indicates the rank of the solution among all others. For each optimal pairs, the sets are ${\mathcal {B}}_t$ and ${\mathcal {S}}_t$ are shown respectively with boxes and lines (“cars”). The height of the rectangle is proportional to the goodness of the solution, in terms of Information Criterion: the best solution has height larger than the second best solution, which in turn has height larger than the third best and so on. This means that *a rule of thumb for choosing the best combination could be to look for the rectangle of largest area*.

Note that, in our approach among all the possible solutions, the best ones are those which are stable along the widest interval of *c* values, and not the solution which maximizes an Information Criterion just for a specific combination of *k* and *c*. It can also happen that rectangles can range through all values of *c* for different values of *k*. In this case, following Occam’s Razor, our preferred solution would be the one associated to the rectangle with smallest *k*, as in the case study presented in Sect. 5.1. It may also happen that the car-bike plot (as in the example of Sect. 4) reveals the presence of more than one solution. In this case we suggest to find the best value of the trimming factor for each of the tentative solutions. The above procedure in the case of TCWM had kept $c_X=128$. For each tentative solution it is possible to investigate the different values of $c_X$ in order to monitor the stability of the results and/or choose the optimal constraint among the scatter matrices in the space of the explanatory variables.

### Estimate of the optimal level of trimming

With the estimated *k* and *c*, we apply TCLUST-REG on the same dataset many times, for different trimming levels $\alpha$. This produces a set of plots for monitoring the change of a series of statistics among two consecutive values of $\alpha$ values, as Figs. 8, 9, 10 and 11 and 18, 19, 20 and 21 show:

- the change in Adjusted Rand Index,
- the change in the regression coefficients. The formula which is used is
  $$\begin{aligned} ||{\hat{b}}_{\alpha _r}-{\hat{b}}_{\alpha _s}||^2/||{\hat{b}}_{\alpha _r}||^2, \end{aligned}$$
  where ${\hat{b}}_{\alpha _r}=vec({\hat{b}}_{1,\alpha _r}, {\hat{b}}_{2,\alpha _r} \ldots , {\hat{b}}_{k,\alpha _r})$ is the column vector of length $p \cdot k$ containing the estimates of the all the regression coefficients for the *k* groups using a trimming level $\alpha _r$, ${\hat{b}}_{j,\alpha _r}= ({\hat{b}}_{0,j,\alpha _r}, {\hat{b}}_{1,j,\alpha _r}, \ldots , {\hat{b}}_{p-1,j,\alpha _r})'$ and symbol *vec* denotes the *vec* operator, while $\alpha _r$ and $\alpha _s$ denote two consecutive levels of trimming ($\alpha _r>\alpha _s$) and *p* is the number of explanatory variables including the intercept. Using squared norm, the computation becomes easier.

#### Remark

given that for each value of the trimming factor the labels of the groups are assigned randomly, we make sure that the labels used were consistent for all values of the trimming factor. More precisely, once the labelling is fixed for the largest value of the trimming factor supplied, we change label *j* into label *i* if:

$$\begin{aligned} \sum _{q=1}^p \left( \frac{ {\hat{b}}_{i,q,\alpha _r}- {\hat{b}}_{j,q,\alpha _s}}{ {\hat{b}}_{i,q,\alpha _r} } \right) ^2 < \min _{l \ne j} \sum _{q=1}^p \left( \frac{ {\hat{b}}_{i,q,\alpha _r}- {\hat{b}}_{l,q,\alpha _s}}{ {\hat{b}}_{i,q,\alpha _r} }\right) ^2. \end{aligned}$$

Groups are successively relabelled in the order of the smallest distance. Note that it may also happen that sometimes the a unique relabelling is not possible in the sense that the new *k* groups are relabelled into $u \le k-2$ groups. In this case our routine produces a warning.^1^

- the change in the error variance.
  $$\begin{aligned} ||{\hat{s}}^2_{\alpha _r}- {\hat{s}}^2_{\alpha _s}||^2/|| {\hat{s}}^2_{\alpha _r}||^2, \end{aligned}$$
  where $s^2_{\alpha _r}=vec({\hat{s}}^2_{1,\alpha _r}, {\hat{s}}^2_{2,\alpha _r}, \ldots , {\hat{s}}^2_{k,\alpha _r})$, is the column vector of length *k* containing the estimate of the error variance for the *k* groups of size $n_1, \ldots , n_k$ using a trimming level $\alpha$. More precisely: ${\hat{s}}_{j,\alpha _r}^2=\sum _{i=1}^{n_j} (y_i-x_i' {\hat{b}}_{j, \alpha _r})^2/n_j$, $j=1, 2, \ldots , k$.

In order to appreciate abrupt change in the estimated error variance, we monitor for each group the values of ${\hat{s}}^2_j$ uncorrected and corrected for truncation as Figs. 9 and 19 show. The correction for truncation keeps into account that the deletion of the $n-h$ most remote observations (where $h=\sum _{j=1}^k n_j$) yields a too small estimates of $\sigma ^2_j$, because it is based on the central *h* observations. The variance of the truncated normal distribution containing the central *h*/*n* portion of the full distribution is:

12 $$\begin{aligned} \sigma ^2(h)=1- \frac{2n}{h} \varPhi ^{-1} \left( \frac{n+h}{2n}\right) \phi \left\{ \varPhi ^{-1}\left( \frac{n+h}{2n} \right) \right\} , \end{aligned}$$

where $\phi (.)$ and $\varPhi (.)$ are respectively the standard normal density and c.d.f. See, for example, (Johnson et al. 1994, pp. 156-162). Therefore, assuming that the groups are subject to the the same level of truncation, the (asymptotically) corrected ${\hat{s}}^{2}_c$ are computed as:

$$\begin{aligned} {\hat{s}}^{2}_{cj}= {\hat{s}}^{2}_j/ \sigma ^2(h). \end{aligned}$$

In order to have an idea of the units which are at boundary among groups and their order of entry into the subset we monitor (see Fig. 20 and the other examples in the Supplementary Material):

- the units which at least once changed allocation or have been trimmed through ad hoc plot (first panel). To simplify the interpretation of the plot, we replaced the group number with card symbols, such as club-suit, diamond-suit, heart-suit, spade-suit; if more groups are needed, then we use other symbols such as circles, bullets, and so on. The changes are permuted in a way that allows to appreciate the relevant groupings, as the clusters of card symbols and unit numbers in purple show;
- the posterior probabilities of each observation with respect to a reference group (second panel).

In order to have an idea of the stability of the fitted values we show (using a colormap) the fitted hyperplanes for each value of $\alpha$ as exemplified by the third panel.

Finally, in order to appreciate the units which are trimmed and the allocation we use a series of subplots which monitor the classification for each value of $\alpha$. The type of plot which is used to monitor the stability of the classification depends on the value of *p*:

1. for $p=1$, we use stacked bars (FSDA function histFS is called);
2. for $p=2$, we use the scatterplot of *y* against the explanatory variable;
3. for $p > 2$, we use partial least square regression and use the scatterplot of *y* against the predictor scores, that is, the first *PLS* component that is linear combination of the variables in *X*. Note that in this way we implicitly can monitor the importance of the various explanatory variables for the classification.

Figure 21 gives an example of the monitoring for a dataset affected by outliers. As the value of $\alpha$ is larger or equal to that of the actual contamination percentage, the classification benefits from trimming, while when the outliers join the subset used for fitting, the statistics show a visible change. More precisely in correspondence of the actual contamination percentage, there is a clear peak in the monitoring of changes of Adjusted Rand Index, regression coefficients and error variances. Similarly, a sudden shift shows up in the monitoring of the error variances for each group or on the fitted regression lines (hyperplanes) of the different groups. Finally, there is a switch of observations from one group to another and a shift in the monitoring of posterior probabilities of each observation. These patterns are discussed in detail in the case study of Sect. 5.2. On the contrary, as the case study of Sect. 5.1 illustrates, peaks, shifts and switches do not appear when data are not affected by outliers.

### Confirmatory forward search

An additional contribution of the paper consists in validating the number of groups *k* estimated in Sect. 3.1. This is done by running the FS (Atkinson and Riani 2004) many times, starting from *R* initial subsets of $m_0$ units randomly chosen, and by monitoring the *R* trajectories of the so called Minimum Deletion Residual (MDR) statistic. Each trajectory is obtained with an iterative progression that computes the regression estimator on *m* units and takes at the next step the $m + 1$ units with smallest squared residuals; the square root of the smallest squared studentized residual among the units not part of this subset is the MDR value. The full MDR trajectory is obtained by iterating from the initial $m=m_0$ to the final $m=n-1$ units. Now, if the dataset contains more groups, the *R* trajectories have the following characteristics:

- Trajectories starting from the same group have similar shape and at some point start to coincide.
- When the units of a group are all included into the subset and the FS progression starts to include the units of a second group, then the MDR suddenly increases forming a peak in the trajectory. With the inclusion of more units of the new group, the MDR slowly decreases, until units from a further group are included producing another upper jump, and so on until all the observations have entered the subset.

Given the above, the MDR trajectories originating from subsets with units in different clusters should be quite separated; in this case the number of groups can be estimated by counting the groups of unique trajectories which are visible in the central part of the plot (see for example Fig. 6).

After discovering the number of groups, it is of interest to verify the quality of the classification. We have developed an approach that alternates (hopefully *k* times) the identification of an homogeneous sub-group using the random start approach and its subsequent elimination, following an idea initially explored in Torti (2011) and Cerioli et al. (2019). This approach replaces the original *k* population (robust) estimation problem with *k* distinct one-population steps, which take advantage of the good breakdown properties of trimmed estimators when the trimming level exceeds the usual bound of 0.5. More precisely, the approach consists in:

- At step *i* ($i=1,\ldots ,k$), generate the MDR plot of the *reduced* dataset cleaned from the observations belonging to groups identified in previous steps.
- Brush the first peak in the trajectories laying outside the bands.
- The group of units associated to the brushed trajectories is automatically identified and removed from the dataset.

Generally, this approach works well in all cases in which the degree of overlapping between the groups is not very high. In the context of international trade, we often have to face with a large proportion of observations falling in a small region near the origin of the axes. The effect of this high-density region can be so strong to override the benefits of trimming and other robust devices, bringing robust techniques, as the random start forward search, to failure (Cerioli and Perrotta 2014).

In this paper, in order to cope with the high density regions, we extend the FS algorithm changing the criterion to select the units which will form the subset at step $m+1$ changes from those which have the smallest squared residuals

$$\begin{aligned} (y_i-x_i^T {\hat{b}}_m)^2 \qquad i=1, 2, \ldots , n \end{aligned}$$

where ${\hat{b}}_m$ is the OLS estimated vector of regression coefficients based on the observations belonging to the subset at step *m*, to those which have the smallest weighted residuals:

13 $$\begin{aligned} (y_i-x_i^T {\hat{b}}_m)^2 w_i \qquad i=1, 2, \ldots , n \end{aligned}$$

This criterion leads us to the following theorem which is proven in the Appendix.

#### Theorem 1

In the context of international trade where we regress value (*V*) again quantity (*Q*) if $w_i$ is given by $1/Q_i^2$, given an estimated price based on *m* observations, the update criterion of Eq. 13 has the interpretation of selecting the $m+1$ transactions which have the closest price to the estimated one.

The new updating scheme increases the probability for high-leverage observations (that is transaction associated with large quantities) to remain in the subset. In other words, this implies that a set of transactions which approximately are located along a particular line, if they are characterized by transactions with large volume and value, will stand out in the random start approach. Next sections will show the benefit of this option.

## A motivating example

The European Commission’s report (Perrotta et al. 2020) has shown how important is to understand the fine-graned structure of the data in the Customs Surveillance system, which centralises all European Union import and export declarations collected from the national customs authorities. For example, during the COVID-19 pandemic that exploded in 2020, the authorities had to study the composition of the EU trade in order to refine the definition of the commodity codes used to import protective equipments (e.g. face masks) from the third countries that can produce them in massive quantities. Thanks to the introduction of the new codes in October 2020, the authorities can now ensure in the EU the supply of protective equipments and other critical commodities. As mentioned in their report, the methods in this article have contributed to this goal.

**Fig. 1:**
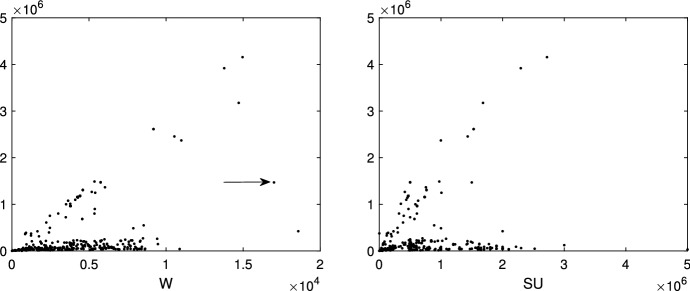
352 imports of FFP2 and FFP3 masks (product 6307909810) into the European Union extracted in a day of November. Vertical axes: traded value, horizontal axes: traded weight (W) and number of units (SU). The point highlighted with an arrow in the left panel is a typical case of a unit which is far from each possible group

The data in Fig. 1 is a sample of 352 import flows extracted in a day of November 2020 from one of the new codes (6307.90.98.10) specific for FFP2 and FFP3 masks. Before this policy decision, a more general product code was covering also other types of face masks (e.g. FFP1) or similar products. For each import flow, we have represented the traded value (vertical axis), weight (“W”, horizontal axis-left panel) and number of units, technically called “Supplementary Units” (“SU”, horizontal axis-right panel).

In this example it is not clear at all how many groups are present, and/or if there are outliers. For example, a transaction is characterized by a combination of quantity and value (17,000–1,468,380) which seems to be very far from the rest. On the other hand, this transaction shows a combination of value and supplementary unit which is much more in agreement with the rest of the population. This transaction has been highlighted with an arrow in the left panel of Fig. 1.

**Fig. 2:**
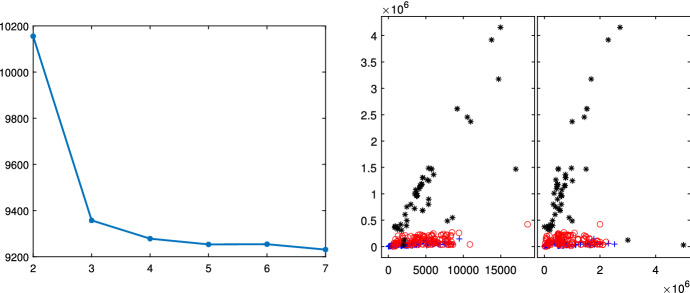
FFP3 and FFP4 imports in November 2020: Flexible Mixture Modeling BIC (left panel) and classification based on $k=4$ (right panel)

**Fig. 3:**
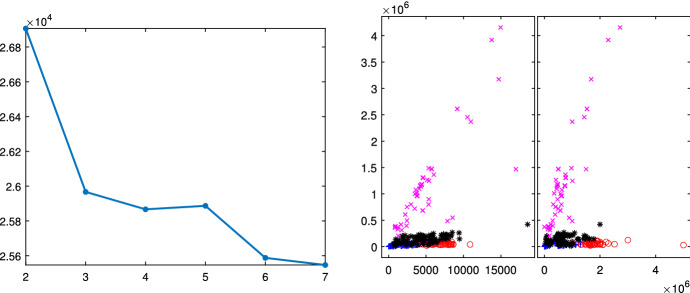
FFP3 and FFP4 imports in November 2020: flexible cluster-weighted modeling BIC (left panel) and classification (right panel)

We have analyzed this dataset using the Flexible Mixture Modeling [R package flexmix (Gruen and Leisch 2007)] and the Flexible Cluster-Weighted Modeling [R package flexCWM (Mazza et al. 2018)]. The results are represented in Figs. 2 and 3 respectively.

The BIC curve of Fig. 2 decreases monotonically; this would indicate that the best number of groups should be as large as possible. We selected as best number of groups 4, where the curve slope starts to be smaller; despite this choice, the Flexible Mixture Modeling identifies only three groups, of which the two on the bottom (red circles and blue crosses) completely overlap. The BIC of Fig. 3 (which is based on the R package flexCWM) shows a local minimum when $k=4$ and for a number of groups larger than 5 it decreases monotonically. This would indicate that the best number of groups should be as large as possible. We therefore selected as best number of groups $k=4$ (value of the local minimum). The plot on the right panel (which contains the associated classification) shows that three of which (red circles, blue crosses and black asterisks) considerably overlap. Moreover, the group of circles is mainly associated with big values of the quantity. It is clear that the complex international trade datasets cannot be analyzed with standard methods, but there is a compelling need of using the tools described in this paper. We will come back to the analysis of this dataset at the end of this paper; in the following sections we show the steps of the suggested procedure using some well known data sets in the regression clustering literature.

## Analysis of selected datasets

**Fig. 4:**
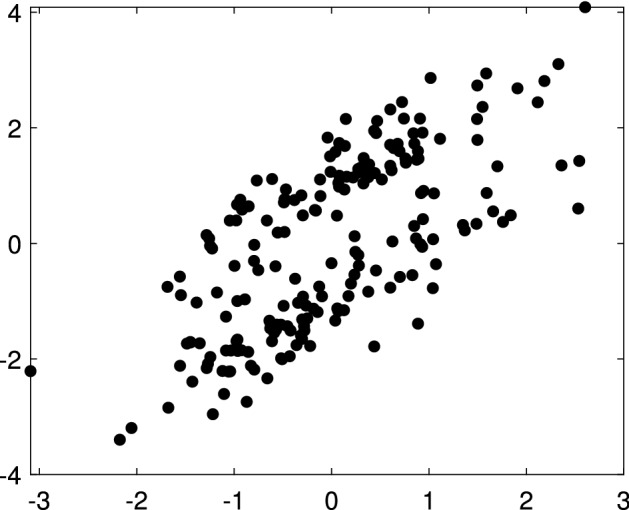
Dataset X: scatterplot

**Fig. 5:**
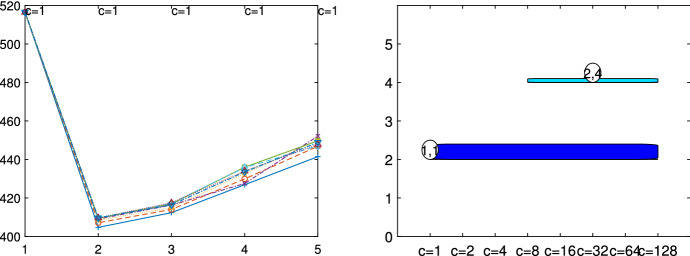
Dataset X: identification of the best number of groups *k* and restriction factor *c* with the elbow plot (on the horizontal axis *k*) and the car-bike plot (on the vertical axis *k*)

**Fig. 6:**
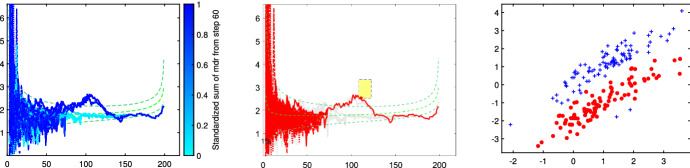
Dataset X: step 1 of iterative FS-based random start approach (the three panels represent respectively steps *a*/*b*/*c* of the iterative approach)

**Fig. 7:**
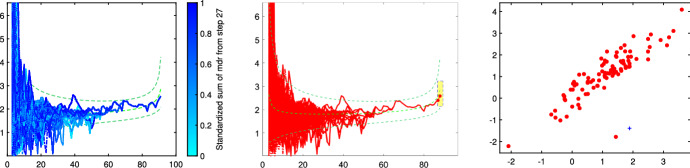
Dataset X: step 2 of iterative FS-based random start approach (the three panels 1/2/3 represent respectively step *a*/*b*/*c* of the iterative approach)

In this section we apply our semiautomatic robust regression clustering tool to a set of real and simulated datasets. In Sects. 5.1 and 5.2 we analyse two well known datasets in the literature. In Sect.  5.3 we analyze the face mask data, while in Sect. 5.4 we concentrate on the analysis of real and simulated international trade data. For all the cases we have applied our procedure using both the TCWM approach and the TCLUST-REG with an adaptive second level trimming. For the datasets in Sects. 5.1 and 5.2 the results remain virtually unchanged. For the international trade datasets the non normality of the data coupled with the fact that in this context high leverage points are highly informative about the characteristics of the different levels of price, much better results are obtained using TCLUST-REG and setting to zero the second level trimming (or a very extreme threshold for adaptive second level trimming). In what follows, for lack of space, we just show the results of the application of TCLUST-REG when we do not apply second trimming level and we use as Information Criterion the Penalized Mixture Likelihood MIX–MIX. The choice of the last information criterion is due to the considerable degree of overlap among the components.

**Fig. 8:**
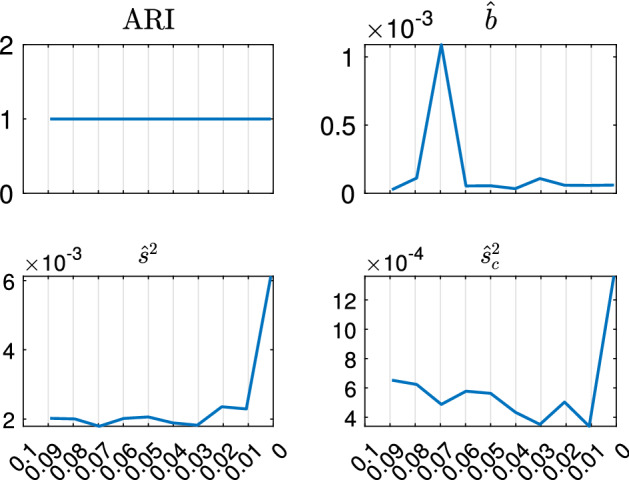
Dataset X: estimation of the best trimming level $\alpha$ (given $k=2$ and $c=1$, identified in Figs. 4, 5, 6 and 7). For 11 values of the trimming level $\alpha$ ranging in the interval [0, 0.1], monitoring of changes in: (i) Adjusted Rand Index, (ii) regression coefficients ${\hat{b}}$ (iii) biased variance ($\hat{s}^2$), (iv) unbiased error variance ($\hat{s}^2_c$)

**Fig. 9:**
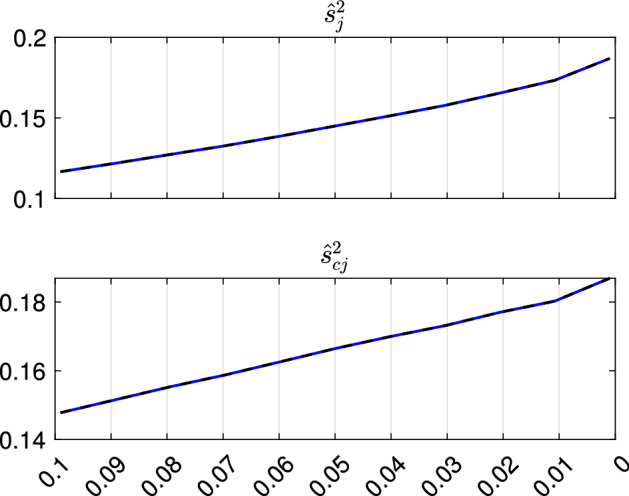
Dataset X: estimation of the best trimming level $\alpha$ (for $k=2$ and $c=1$, identified in Figs. 4, 5, 6 and 7). For 11 values of the trimming level $\alpha$ ranging in the interval [0, 0.1], monitoring for each group (i) error variances ($\hat{s}^2_j$), (ii) unbiased error variances ($\hat{s}^2_{cj}$)

**Fig. 10:**
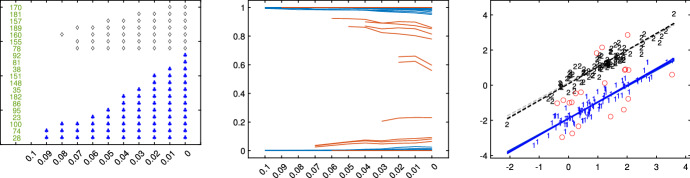
Dataset X: estimation of the best trimming level $\alpha$ (given $k=2$ and $c=1$, identified in Figs. 4, 5, 6 and 7). For 11 values of the trimming level $\alpha$ ranging in the interval [0, 0.1], monitoring: *1st panel*: units changing classification; *2nd panel*: posterior probabilities of each unit; *3rd panel*: scatter of the *k* groups with the $11 \times k$ regression lines

**Fig. 11:**
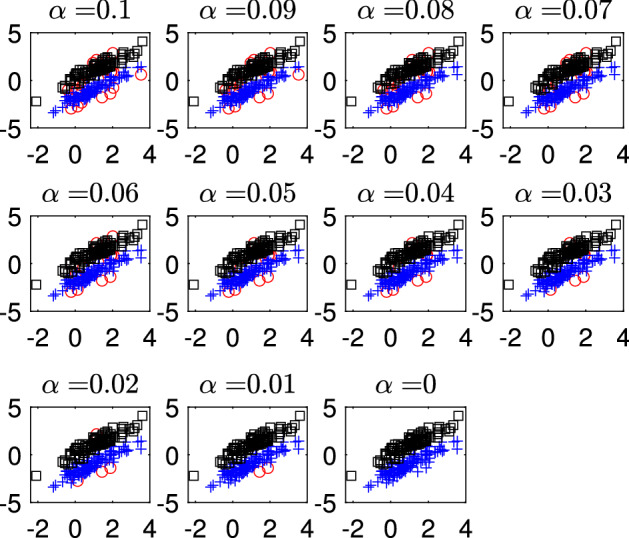
Dataset X: estimation of the best trimming level $\alpha$ (given $k=2$ and $c=1$, identified in Figs. 4, 5, 6 and 7). For 11 values of the trimming level $\alpha$ ranging in the interval [0, 0.1], monitoring the scatter of *y* vs *X* with allocation for each of the 11 values of $\alpha$

### X data

The X dataset, shown in Fig. 4, was simulated by Alfonso Gordaliza, Luis Angel García-Escudero and Agustin Mayo-Iscar during the Workshop “Advances in robust data analysis and clustering” held in Ispra on October 21st-25th 2013. It is is a bivariate dataset of 200 observations, with two parallel components without contamination^2^. Figures 5, 6 and 7 study the choice of *k* and *c* with the elbow plot, the minimum deletion residual plot and the car-bike plot. The elbow plot (Fig. 5, left panel) shows for some combinations of *k* and *c* the mixture likelihood applied on a mixture model, which is equivalent to the Bayesian Information Criterion (BIC). At the top of the plot we show the best value of *c* for each *k*. For example, in this case the plot clearly suggests as best combination $k=2$ and $c=1$. The car-bike plot (Fig. 5, right panel) shows that there are just two different solutions one with $k=2$ which is stable and best for all values of *c* and the other with $k=4$. The first solution is to be preferred in terms of BIC, stability and higher rectangle area. The minimum deletion residual plot (Fig. 6, left panel) shows that, independently from where the search starts, all the trajectories collapse in two main ones, indicating therefore the presence of two groups. With the iterative procedure described in Sect. 3.3, by brushing the first peak outside the envelopes, as shown in correspondence of the yellow rectangle in the central panel of Fig. 6, we identify the first group. The trajectory based on the remaining observations (7), which form only one group, lies inside the envelopes; by brushing it anywhere on the right of the point of convergence, we identify the second group.

Figures 8, 9, 10 and 11 study the choice of the trimming level $\alpha$ of TCLUST, with a number of visualization instruments. Following the results above, the analysis has been conducted for $k=2$ groups and $c=1$ restriction factor. All the resulting plots brings to the conclusion that the trimming level $\alpha$ does not have any influence on the final classification. In Fig. 8, the changes in Adjusted Rand index, the regression coefficient, and errors variance have very small values ($10^{-3}$, $10^{-3}$ and $10^{-4}$ respectively). The peaks that show up are therefore irrelevant. In Fig. 9 the variances and unbiased variances of the different groups coincide (given that in this case the best restriction factor *c* is equal to 1) and increase gently as the trimming level $\alpha$ decreases (as expected) without sudden peaks. The clear separation between the symbols associated with the two groups in the left panel of Fig. 10 indicates that there is no interchange of observations from one group to another when the values of $\alpha$ change: the trimming level does not have any influence on the final classification. The posterior probabilities of each observation for different trimming levels (central panel of Fig. 10) are divided into two well separated groups and there are no trajectories of units that change assignment for different $\alpha$ levels. The right panel of Fig. 10 shows that for each group the regression lines obtained with different levels of $\alpha$ almost overlap. Finally, Fig. 11 shows that the level of $\alpha$ does not have any influence on the scatters which show the allocation to the two groups.

### Pinus data

**Fig. 12:**
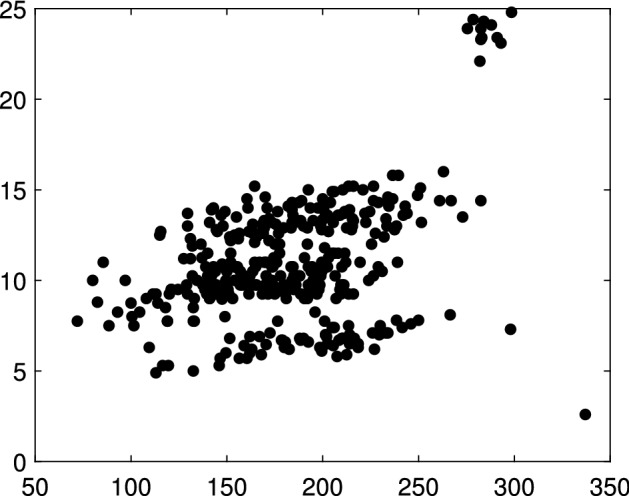
Pinus dataset: height (*y* axis, in meters) and diameter (*x* axis, in millimeters) of 362 Pinus nigra trees located in the north of Palencia (Spain)

The pinus dataset, represented in Fig. 12, was introduced by García-Escudero et al. (2010a) and further discussed by Dotto et al. (2017) and Greco et al. (2020). It consists of the heights and diameters of a sample of 362 pinus nigra trees, located in the north of Palencia (Spain). The scatterplot of this bivariate dataset clearly shows the presence of three linear groups apart from a small group of trees forming its own cluster on the top right corner and one isolated point on the bottom right corner.

**Fig. 13:**
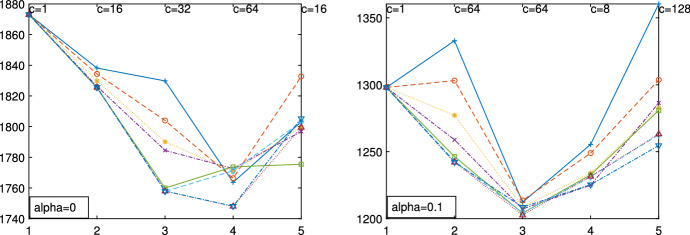
Pinus dataset: elbow plot (on the horizontal axis *k*) obtained for $\alpha =0$ and $\alpha =0.1$ respectively

**Fig. 14:**
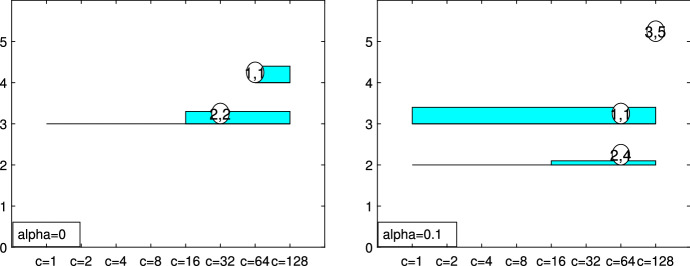
Pinus dataset: car-bike plot (on the vertical axis *k*) obtained for $\alpha =0$ and $\alpha =0.1$ respectively

**Fig. 15:**
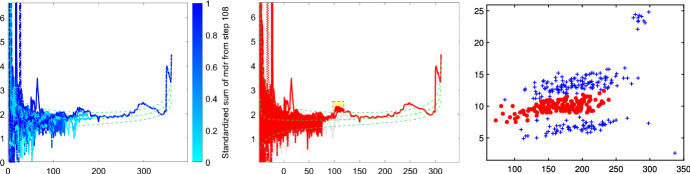
Pinus dataset: cluster identified by brushing the minimum deletion residual plot computed on all observations

**Fig. 16:**
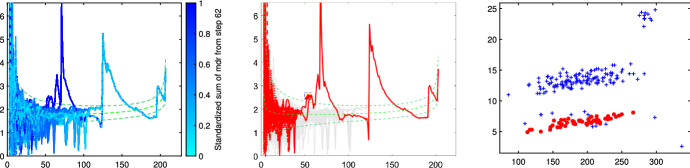
Pinus dataset: cluster identified by brushing the minimum deletion residual plot computed on all observations after having excluded the cluster identified in Fig. 15

**Fig. 17:**
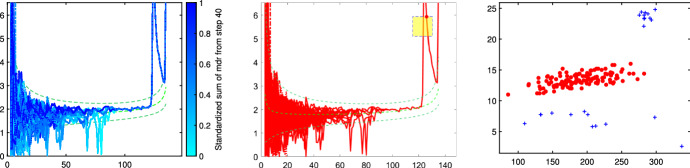
Pinus dataset: cluster identified by brushing the minimum deletion residual plot computed on all observations after having excluded the clusters identified in Figs. 15 and 16

Figures 13, 14, 15, 16 and 17 study the choice of *k* and *c* with the elbow plot, the car-bike plot and the minimum deletion residual plot. In the previous example the results of using $\alpha =0$ were equal to those with $\alpha =0.10$. On the other hand, in this case, the choice of the optimal values of *k* and *c* depend on the level of trimming which is used. In particular for the no-trimming case (left panel of Fig. 13), suggested solution is $[k=3,c=32]$ or $[k=4,c=64]$; when $\alpha =0.1$, on the other hand (right panel of Fig. 13) a possible solution could be $[k=3,c=64]$. The two car-bike plots (14) seem to indicate that the best solution is $k=3$ with a positive first level trimming. The minimum deletion residual plot does not clearly show the *k* trajectories. However the iterative random start approach, described in Sect. 3.3, allows to identify three groups (Figs.  15, 16 and 17). In summary, the joint analysis coming from the different tools seems to indicate the presence of 3 groups, $c=64$ and the need of trimming.

**Fig. 18:**
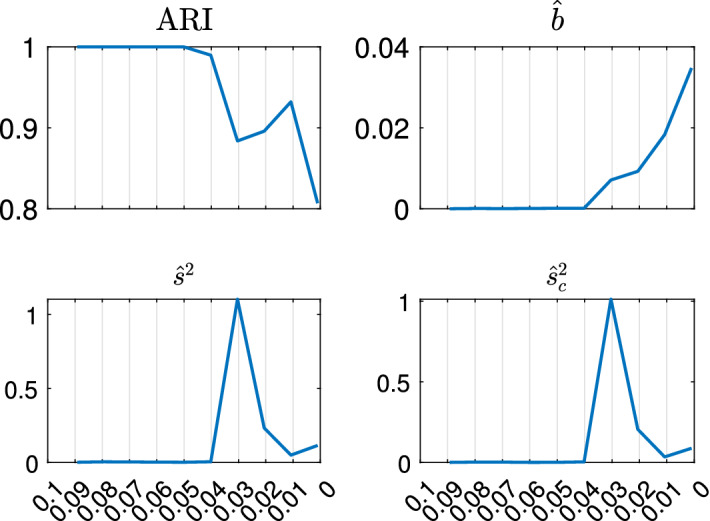
Pinus dataset: estimation of the best trimming level $\alpha$ (given $k=3$ and $c=8$, identified in Figs. 13, 14, 15, 16 and 17). For 11 values of the trimming level $\alpha$ ranging in the interval [0, 0.1], monitoring of the changes in (i) Adjusted Rand Index , (ii) regression coefficients $\hat{\beta }$, (iii) error variance ($\hat{s}^2$), (iv) corrected error variance ($\hat{s}^2_c$)

**Fig. 19:**
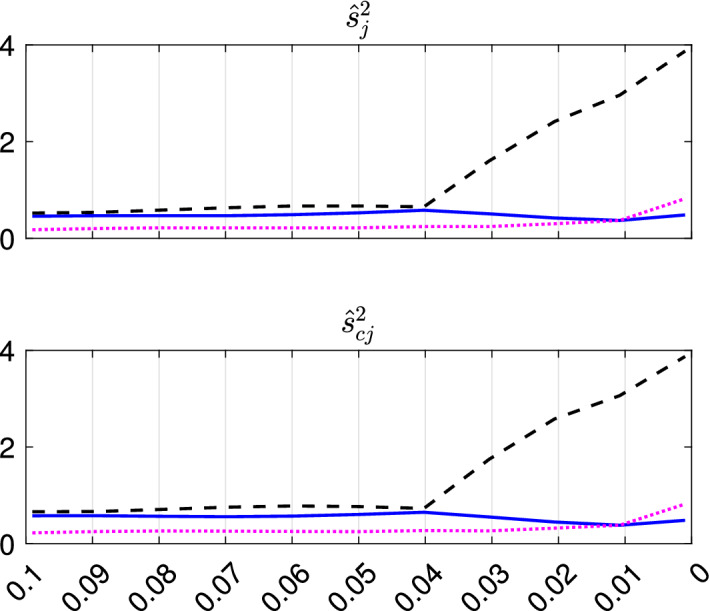
Pinus dataset: estimation of the best trimming level $\alpha$ (given $k=3$ and $c=8$, identified in Figs. 13, 14, 15, 16 and 17). For 11 values of the trimming level $\alpha$ ranging in the interval [0, 0.1], monitoring for each group (i) error variances ($\hat{s}^2_j$), (ii) unbiased error variances ($\hat{s}^2_{cj}$)

**Fig. 20:**
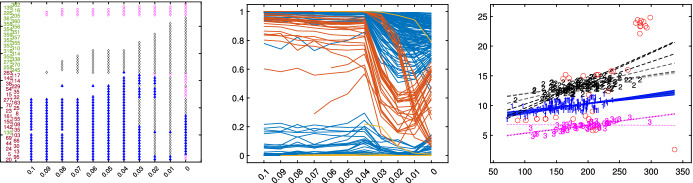
Pinus dataset: estimation of the best trimming level $\alpha$ (given $k=3$ and $c=8$ identified in Figs. 13, 14, 15, 16 and 17). For 11 values of the trimming level $\alpha$ ranging in the interval [0, 0.1], monitoring of the: *1st panel*: units changing classification; *2nd panel*: posterior probabilities of each unit. *3rd panel*: scatter of the *k* groups with the $11 \cdot k$ regression lines

**Fig. 21:**
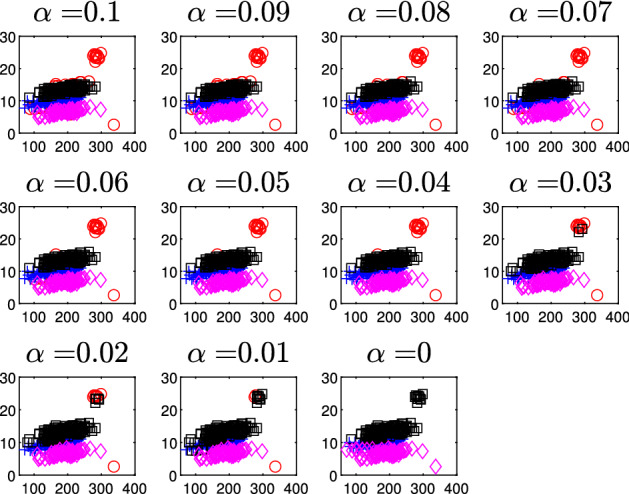
Pinus dataset: estimation of the best trimming level $\alpha$ (given $k=3$ and $c=8$ identified in Figs. 13, 14, 15, 16 and 17). For 11 values of the trimming level $\alpha$ ranging in the interval [0, 0.1], monitoring of the scatter of *y* against *X* with allocation for the 11 values of $\alpha$ ranging in the interval [0, 0.1]

Figures 18, 19, 20 and 21 study the choice of $\alpha$. All the plots in these figures bring to the conclusion that the estimates are stable up to trimming level $\alpha \ge 0.04$. This is evident in the monitoring of the changes in ARI index, regression coefficient, and global error variance (Fig. 18) or in the monitoring of the variances ${\hat{s}}^2_j$ of the three groups (Fig. 19). The clear separation among the symbols associated to the three groups for $\alpha \ge 0.04$ and their shuffling for $\alpha < 0.04$ in the first panel of Fig. 20 indicates that $\alpha =0.04$ is the minimum trimming level for having stable solutions. The same conclusion can be drawn by analysing the posterior probabilities of units changing groups, represented by red trajectories, in the second panel: the main modifications in the trajectories appear in correspondence of $\alpha < 0.04$. The right panel shows that for group number 1 the regression lines obtained with different levels of $\alpha$ almost overlap; but this is not the case for group numbers 2 and 3 which are affected by the outliers when the trimming level is too small. Finally the scatter of *y* against *X* for each value of $\alpha$ in Fig. 21 shows that the outliers are correctly trimmed when $\alpha \ge 0.04$. All the plots in this case show that the optimal level of trimming is $\alpha =0.04$.

The results above are stable if we impose the constraint of common slope. More specifically, with this constraint the value of the common slope we obtain ${\hat{b}}_{1,\alpha =0.04}=0.0151$ which is exactly equal to the value obtained by Greco et al. (2020). The complete set of coefficients is shown in Table 1.

**Table 1:** Pinus data: estimated coefficients, when $\alpha =0.04$ and $k=3$

Common slope			
${\hat{b}}_{0,\alpha =0.04}$	3.687	7.3802	10.5371
${\hat{b}}_{1,\alpha =0.04}$	0.0151		
*Different slopes*			
${\hat{b}}_{0,\alpha =0.04}$	3.9336	7.4278	10.3572
${\hat{b}}_{1,\alpha =0.04}$	0.0138	0.0148	0.016

### Analysis of face masks data

**Fig. 22:**
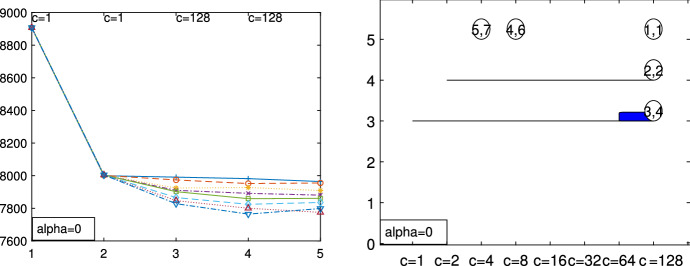
Face masks data: elbow plot (*k* on the horizontal axis) and car-bike plot (*k* on the vertical axis)

In this Section we present the analysis of imports of FFP3 and FFP4 face masks introduced in Sect. 4. The left panel of Fig. 22 (elbow plot) shows that the best solution suggested by the information criterion is in correspondence of $c=128$ and $k=5$. On the other hand, the car-bike plot in the right panel of Fig. 22, indicates that this solution is very local, being valid just for this (*c*, *k*) combination. The plot also shows two solutions for $k=3$ and $k=4$ which deserve particular attention.

**Fig. 23:**
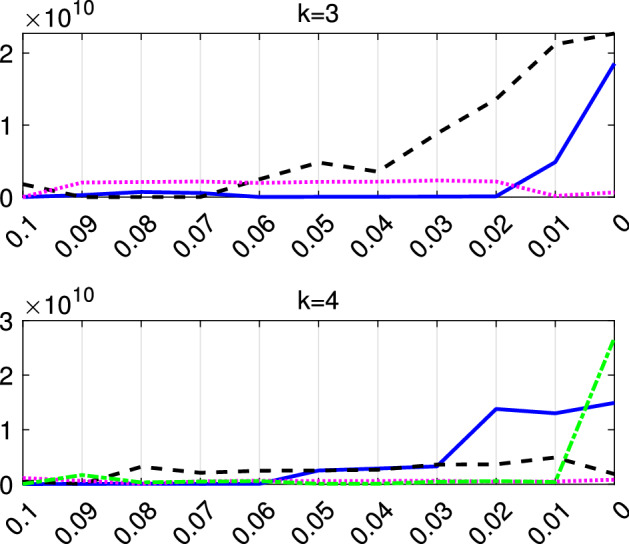
Face masks data: monitoring of error variances when $k=3$ (top panel) and $k=4$ (bottom panel)

**Fig. 24:**
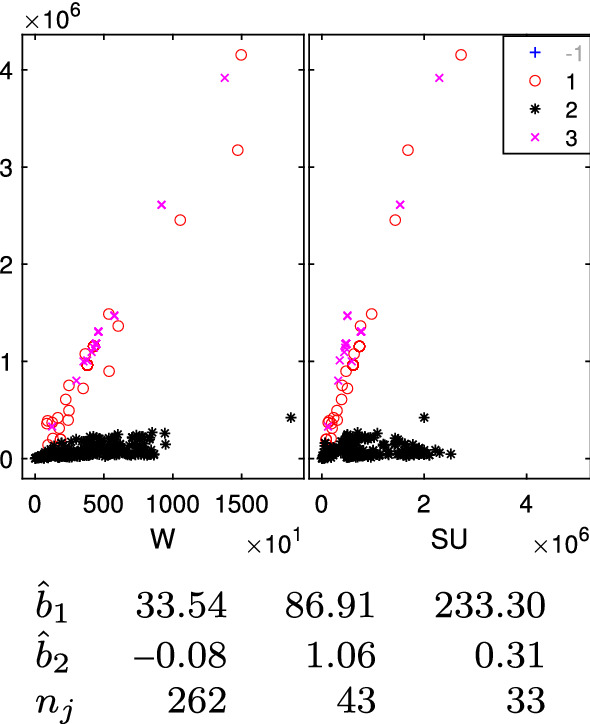
Face mask data: final classification based on $k=3$; the $4\%$ trimmed units (denoted in the legend with symbol ‘+’ -1 in faint grey) are not shown

**Fig. 25:**
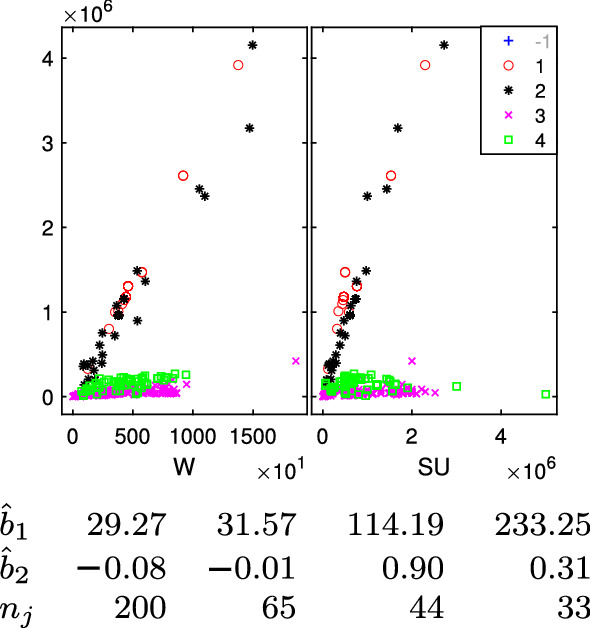
Face mask data: final classification based on $k=4$; the $3\%$ trimmed units (denoted in the legend with symbol ‘+’ -1 in faint grey) are not shown

The monitoring of each group error variance $\hat{s}^2_j$ for $k=3$ shows a clear increase when $\alpha =0.03$. On the other hand, when $k=4$ the big increase takes place when $\alpha =0.02$. Therefore, the optimal levels of trimming are 0.04 and 0.03 respectively. Figures 24 and  25 report the final TCLUST classifications of the good units (the untrimmed ones are not shown) together with the estimated regression coefficients and the associated group sizes. In both cases, the data appear partitioned in very sensible groups, which capture the fine grained structure of this (only apparently simple) dataset. The slope (${\hat{b}}_{1,l}$) coefficients, which represent the estimated prices per Kg, can be compared to those found by Perrotta et al. (2020) for 5 groups using the data that were available before the definition of the new codes: 2.77, 26.21, 40.77, 93.11, 169.18 euro per Kg.

We conclude the analysis of this example comparing the degree of internal cohesion inside each group in the final classification between the suggested approach and the output from flexmix and flexCWM (as described in Sect. 4). Table 2 contains the value of adjusted $R^2$ (in non decreasing order) inside each group for the different methods. The different degree of homogeneity inside each group is very evident.

**Table 2:** Adjusted $R^2$ inside each group in non decreasing order

3 Groups	4 Groups	flexmix	flexCWM
0.9999	0.9999	0.936	0.9425
0.9933	0.9909	0.7962	0.6698
0.5376	0.7849	0.3914	0.3813
	0.5074	– 0.0296	

First two columns: our final classification based on 3 and 4 groups. Third and fourth column: classification from flexmix and flexCWM

From the comparison of the two sets of regression coefficients, we conclude that the introduction of the new code has changed the structure of the data declared by traders in relation to FFP2 and FFP3 face masks. In fact, with the new more specific codes, the cheapest group (2.77 euro per Kg) disappears and a new highly priced group appears (233 euro per Kg). This indicates that, as expected, the FFP2 and FFP3 masks are the most expensive category of masks, but also that the prices have increased from the beginning of the COVID crisis.

### Simulated trade-like data

**Fig. 26:**
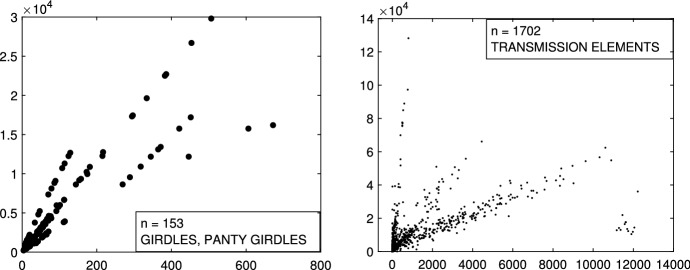
Real trade data. *First panel*: 153 imports of girdles and panty girdles (product code 6212.20.00.00 in the combined international nomenclature) from a given third country to a specific Member State. *Second panel*: 1702 imports of toothed wheels, chain sprockets and other transmission elements (product code 8483.90.89.90 in the combined international nomenclature) from a given third country to a specific Member State

**Fig. 27:**
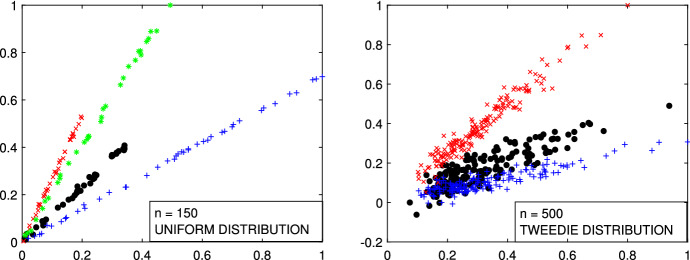
Simulated trade like data where the X variable is simulated from a Uniform (*1st panel*) and from a Tweedie (*2nd panel)*

In this Section we focus our attention to additional international trade data, that the Joint Research Centre of the European Commission routinely analyzes in order to estimate fair prices and detect potential fraudulent behaviour. These datasets are very different one to the other and they always have structures with high level of complexity: in particular they can be characterized by extreme collinearity on one hand or extreme dispersion on the other hand. In the first panel of Fig. 26, there is an example of trade data with 3 extremely collinear groups. On the vertical and horizontal axes there are respectively the traded value and weight of 153 imports of a textile products from a given third country to a specific Member State. The second panel gives an example of trade data with 3 extremely disperse groups. It contains 1702 import flows of mechanical parts from a given third country to a specific Member State. In international trade data, in general we do not have a test set with the real classification. Therefore in order to study the performances of our techniques we simulate trade like data containing the typical trade data patterns. For example the two simulated data represented in Fig. 27 mimic the two trade data in Fig. 26. They were simulated using MIXSIM (Maitra and Melnykov 2010; Riani et al. 2015), with the independent variable distributed as Uniform and Tweedie respectively. The motivation for the use of the Tweedie distribution is discussed in Barabesi et al. (2016). The analysis of all these data, together with a comparison with existing methods, is available in the Supplementary Material.

## Conclusions

The detection of fraudulent behavior in economic activities has recently assumed a key role in the political priorities of the European Commission. In this paper we have illustrated a series of methods for the semiautomatic classifications of the observations. More in detail, while it is by now well accepted that unsupervised classification problems have to be addressed using a robust approach, it is not clear how to automatically choose the number of components, the optimal level of restriction factor to impose among the residual variances across the groups and the optimal level of trimming. For example, imposing a level of trimming smaller than optimal can lead to masking problems, while overtrimming has the drawback of obtaining estimates which are not efficient. In this paper we have given a series of guidelines for the semiautomatic choice of the number of regression hyperplanes, optimal level of trimming and restriction factor. We have also proposed a new way of selecting units during the progression of the random starts forward search which, in the context of international trade data, has a sound motivation and interpretation. The results have been shown using simulated and real datasets which have different level of contamination, different degree of heavy tails and different variability across groups. All the results obtained are easily reproducible because they use downloadable code coming from a public repository in github.

Clearly not all trimmed units are associated with “fraudulent behaviour” . Sometimes, for example in the context of international trade the different strips can be attributed to external “normal “factors which cannot be captured by the simple analysis of quantity (*Q*), values (*V*) and supplementary units. It will be of paramount importance in the future the setting up of an integrated system which, not only analyzes the quantitative data referred to traded weight, value and number of units, but also pays attention to the plethora of qualitative information which is present in the original single administrative documents which characterize all the imports. A frequent suggestion that we receive from reviewers is to analyse data after applying a log transformation. This option sometimes simplifies the estimation of the number of groups but, when the number of observations in each clusters is very unbalanced, the introduction of the additional parameter of the intercept (necessary for the log transformation) makes the estimation very unstable. On the other hand, if the model is forced to pass through the origin, components with few observations are more easily identified. In addition, if the estimated slope in a log transformed model is significantly different than $\pi /2$, the interpretation of the results in term of price is not trivial. Given that the ultimate beneficiary of this type of trade data analysis is the anti-fraud investigator, it is important to grant the maximum interpretability and avoid introducing complications that are difficult to bring in front of a Court.

## Appendix

In this appendix we prove Theorem 1. In the context of international trade data we regress $V_i=p_i \times Q_i$ on $Q_i$. The estimate of the price ${\hat{p}}_m$ given a subset $S_m$ of size $m \le n$ is given by

$$\begin{aligned} {\hat{p}}_m = \frac{ \sum _{i \in S_m} p_i Q_i}{ \sum _{i \in S_m} Q_i^2} \end{aligned}$$

The residuals for all the observations weighted with the inverse of the quantity:

$$\begin{aligned} (V_i - {\hat{p}}_m Q_i )^2 \frac{1}{ Q_i^2} \qquad i=1, 2, \ldots , n \end{aligned}$$

can be rewritten as:

$$\begin{aligned} \left( \frac{V_i}{Q_i} - {\hat{p}}_m \right) ^2 \qquad i=1, 2, \ldots , n \end{aligned}$$

or

$$\begin{aligned} (p_i - {\hat{p}}_m )^2 \qquad i=1, 2, \ldots , n. \end{aligned}$$

Selecting as next subsets the units with the smallest $m+1$ squared residuals therefore, is equivalent to select the $m+1$ transactions which have the minimum distance with ${\hat{p}}_m$.

## Footnotes

1 Additional details can be found directly from the help file of the routine at the web address http://rosa.unipr.it/FSDA/clusterrelabel.html.

2 All the datasets in this paper are included in the FSDA toolbox in the section clustering datasets.
